# Oligosaccharide feed supplementation reduces plasma insulin in geldings with Equine Metabolic Syndrome

**DOI:** 10.3389/frmbi.2023.1194705

**Published:** 2023-08-02

**Authors:** Alexandra von Münchow, Sarah Torp Yttergren, Rasmus Riemer Jakobsen, Nanna Luthersson, Axel Kornerup Hansen, Frederikke Lindenberg

**Affiliations:** ^1^ Section of Experimental Animal Models, Department of Veterinary and Animal Sciences, Faculty of Health and Medical Sciences, University of Copenhagen, Copenhagen, Denmark; ^2^ Hestedoktoren, Højgaard Sjælland ApS, Hvalsoe, Denmark; ^3^ Section of Microbiology and Fermentation, Department of Food Sciences, Faculty of Science, University of Copenhagen, Copenhagen, Denmark; ^4^ Brogaarden ApS, Lynge, Denmark

**Keywords:** Equine Metabolic Syndrome, prebiotics, oligosaccharide, fatty acids, volatile, gut microbiota, microbiology

## Abstract

**Background:**

The prevalence of Equine Metabolic Syndrome and insulin dysregulation is increasing in the horse population. Insulin dysregulation is a risk factor for laminitis, which has significant welfare consequences for the horse. Current treatment strategies for Equine Metabolic Syndrome have variable effects on insulin sensitivity. Findings suggest that gut microbiota plays an important role in gastrointestinal barrier protection and metabolic diseases. Dietary supplementation with oligosaccharides has been shown to increase the production of short-chain fatty acids and induce changes in the gut microbiota, with an increase in insulin sensitivity.

**Objectives:**

We hypothesized that dietary oligosaccharide supplementation would ameliorate insulin dysregulation in horses with Equine Metabolic Syndrome.

**Study design:**

Fifteen horses were included in a cross-over study including two feed supplementations periods with oligosaccharide supplementation or calcium carbonate. Before and after each period plasma insulin, endotoxin, serum amyloid A, and blood glucose concentrations were measured during an oral sugar test and fecal samples were collected.

**Results:**

Oligosaccharide supplementation significantly reduced insulin in geldings (*p* = 0.02). Overall, none of the dominating bacterias changed significantly in relative Δ-abundance. In the gut microbiota of mares, the *Akkermansia* genus and *Clostridiaceae* family were significantly more abundant. Within-sample bacterial diversity of horses with insulin concentrations > 60 mIU/L was significantly greater when compared to horses < 60 mIU/L. Horses with insulin concentrations > 60 mIU/L and horses with previous laminitis had a significantly greater beta diversity.

**Main limitations:**

Cut-off values for oligosaccharide feed supplementation/placebo dosing instead of dosing by g/kg body weight; owner compliance; single blood sample in oral sugar test; inter-horse variation

**Conclusions:**

This study demonstrated that oligosaccharide supplementation could improve insulin dysregulation in geldings with Equine Metabolic Syndrome suggesting that sex susceptibility might be a factor to consider. Additionally, a tendency of reduction of obesity-associated bacteria was observed. Furthermore, the diversity of the gut microbiota was significantly dependent on laminitis status and insulin concentrations. However, more studies on the effects of oligosaccharide supplementation on insulin dysregulation are needed to improve the dietary management of horses with Equine Metabolic Syndrome.

## 1 Introduction

Equine Metabolic Syndrome (EMS) is an increasingly common clinical syndrome in horses, often predisposed by a positive energy balance, inadequate physical activity, and obesity (Treiber et al., 2006; Carter et al., 2009; Frank et al., 2010). Equine obesity is a wide-spanning problem, and approximately 45% of the horse population evaluated in the United Kingdom were obese (Wyse et al., 2008). Equine Metabolic Syndrome is a collection of risk factors for endocrinopathic laminitis (Durham et al., 2019). The most important is insulin dysregulation (ID), which can manifest as hyperinsulinemia and/or insulin resistance (IR) (de Laat et al., 2016). Laminitis has significant welfare and economic consequences; still, no adequate treatment is available, and laminitis is associated with decreased survival after one year of onset (McGowan et al., 2004). Treatment strategies for ID are based on weight reduction (Van Weyenberg S et al., 2007) and exercise (McCutcheon et al., 2002; Harris et al., 2006; Stewart-Hunt et al., 2006; Liburt et al., 2010; Bonelli et al., 2017), the latter being non-applicable in horses with current or recent laminitis (Durham et al., 2019). Metformin hydrochloride (MH) is commonly used but has poor oral bioavailability in horses (Hustace et al., 2009) with variable effects on insulin sensitivity (SI) (Durham et al., 2008; Tinworth et al., 2012). Hence, new management strategies to counter ID are needed.

The EMS phenotype is dominated by obesity and/or regional adiposity, often around the nuchal ligament (“cresty neck”) and tail head (Frank et al., 2010). In studies analyzing the gut microbiota (GM) composition of obese horses (Biddle et al., 2018; Morrison et al., 2018) and mice (Marchesi et al., 2015), a significantly increased relative abundance of bacteria within the Firmicutes phylum and especially *Lactobacillus* spp. (species) have been observed. The human adult GM consists of 90% Firmicutes and Bacteroidetes phylum, which contributes to the regulation of the immune system by improving the gut barrier function. This is done by competing with pathogenic bacteria for nutrients (Brahe et al., 2016). Adipose tissue is an endocrine organ, and the equine “cresty neck” might be an especially active depot (Burns et al., 2010). Important adipose tissue factors are proinflammatory cytokines (Guilherme et al., 2008; Tsatsoulis et al., 2013; Ye, 2013). In mice, proinflammatory cytokines may contribute to intestinal barrier dysfunction (Brun et al., 2007), and obese horses had a higher endotoxin flux across the mucosa of the jejunum compared to lean horses (Kopper et al., 2019). Endotoxins is a virulence factor from gram negative bacteria in the gut (Pussinen et al., 2011). Human metabolic syndrome is characterized by a systemic inflammation caused by increased systemic endotoxin concentrations (Pussinen et al., 2011). Equine and human metabolic syndrome share similarities in the systemic inflammation (Ertelt et al., 2014). Obesity and a positive energy balance have been associated with altered GM and increased intestinal permeability, causing increased endotoxin concentrations. Systemic endotoxin blocks the insulin-mediated glucose uptake in cells generating high systemic glucose concentrations characterizing metabolic syndrome (Lindenberg FCB. et al., 2019). Thus, there are indications that obesity and/or ID increase the risk of intestinal translocation of bacterial endotoxins despite an increased abundance of Firmicutes (Kopper et al., 2019). In humans (Dao et al., 2016) and rodents (Everard et al., 2013; Shin et al., 2014; Anhe et al., 2015), an association between GM composition and SI has been suggested, with a positive correlation between the abundance of *Akkermansia muciniphila* and health (Cani and de Vos, 2017). *Akkermansia muciniphila* is a Gram-negative, anaerobe mucin-degrading bacterium of the *Verrucomicrobia* phylum (Collado et al., 2007). It produces short-chain fatty acids, which act as substrates for other microorganisms and the host (Collado et al., 2007; Everard et al., 2013; Lukovac et al., 2014; Shin et al., 2014; Anhe et al., 2015; Dao et al., 2016). In horses, *Clostridiales* spp. and *Verrucomicrobia* spp. were positively correlated to increased expression of the regulatory T cell transcription factor in the ileum and cecum. Hence, species such as *Akkermansia muciniphila* can potentially induce a regulatory immunity (Lindenberg F. et al., 2019) which might downregulate inflammation caused by obesity (Cani et al., 2012).

Lindenberg et al. (2021) showed that a dietary supplement with mannan- and fructo-oligosaccharides changed the GM of 49 days old foals, with a significant increase in the abundance of *Akkermansia* spp (Lindenberg et al., 2021). Soluble fibres in the diet of horses also increased caecal short-chain fatty acids and plasma propionic acid concentrations, resulting in stable glycemic and insulinemic responses (Brokner et al., 2016). Short-chain fatty acids produced by the GM are the main nutrients for colonocytes (Chan, 2013) and strengthen gut barrier function by regulation of intestinal mucin (Brahe et al., 2016). Respondek et al. (2011) found that a six-week supplementation with short-chain fructo-oligosaccharides (sc-FOS) (45 g/daily) in eight Arabian geldings was associated with increased SI (Respondek et al., 2011). However, a similar study by McGowan et al. (2013) in 10 mares and two geldings of varying breeds did not find an additional effect of 10 g sc-FOS/100 kg/daily (McGowan et al., 2013), suggesting sex or breed susceptibility to the effects of prebiotics (McGowan et al., 2013).

The purpose of this study was to examine the effects of oligosaccharide feed supplementation (OS) on ID in horses diagnosed with or matching the phenotype for EMS. Plasma insulin concentration was chosen as primary readout, while secondary readouts were glucose, endotoxin, serum amyloid A (SAA), fecal differential abundance analysis, Shannon diversity analysis, and beta diversity analysis. We hypothesized that circulating insulin would significantly decrease in the group receiving OS compared to the control group. Further, we sought to investigate differences based on sex, age, breed, insulin concentrations, current MH treatment, and prior laminitis diagnosis.

## 2 Materials and methods

### 2.1 Ethics

Oral and written approval was obtained from all owners before the study. The performed study was approved by the Animal Ethics Institutional Board (Animal Ethics Institutional Board, Department of Veterinary and Animal Sciences, Faculty of Health and Medical Sciences, University of Copenhagen) with approval number 2021-04-EAM-015A.

### 2.2 Animals

Fifteen privately owned horses including four ponies were used in this study. Inclusion criteria were body condition score (BCS) ≥ 5 and a veterinary history diagnosing EMS or a phenotype suspecting EMS (see **Appendix 1**). Adiposity was assessed by the presence of a “cresty neck” and with BCS evaluated on a nine-point scale defined by Henneke et al. (1983) (Henneke et al., 1983). Areas palpated were ribs, tailhead, spinous processes, and neck. Age varied between 3 and 19 years, with a mean of 10.9 years, and weight ranged between 138 kg and 640 kg on the day of the first oral sugar test (OST) (see **Appendix 2**). Body condition score varied between 6/9 and 8/9, with a mean of 6.8. Fourteen of the 15 horses had a cresty neck. Eight horses had previously been diagnosed with ID, with insulin levels > 60 mIU/L following an OST (Lindase et al., 2016). Five of the horses diagnosed with ID were medically treated with MH (horses 2, 8, 9, 10, and 15; **Table 1**) at study initiation. Subsequently, two horses had MH treatment withdrawn during the wash-out period after veterinary consideration - one after OS (horse 8; **Table 1**) and the other after placebo treatment (horse 15; **Table 1**). Two horses had prior to this study been diagnosed with pituitary pars intermedia dysfunction (PPID) (horse 10 and 15; **Table 1**). Five out of 15 horses were previously diagnosed with laminitis (horses 2, 8, 9, 11, and 15; **Table 1**). One horse became temporarily lame during the wash-out period, however, without clinical or radiographic signs of laminitis. Another horse was diagnosed with Polysaccharide Storage Myopathy. All horses remained in the study. Horses were kept at their respective housing facility throughout the experiment (see **Appendix 3**). All owners agreed to maintain the same individual feed type and volume, housing, and paddock time and type throughout the entire study.

**Table 1:** Fifteen horses were included in a cross-over study at week 0.

Number	Sex	Gelding	Age (years)	Breed	Group	BCS (1–9)	Cresty neck (yes/no)
1	Female	–	12	Fjord horse	G2	7	Yes
2	Female	–	7	Icelandic horse	G1	6	Yes
3	Female	–	9	Shetland pony	G1	6	Yes
4	Female	–	3	Frederiksborg horse	G1	8	Yes
5	Female	–	5	Frederiksborg horse	G2	8	Yes
6	Female	–	16	Shetland pony	G2	7	Yes
7	Female	–	18	Shetland pony	G1	6	Yes
8	Male	Yes	14	Crossbreed	G1	7	Yes
9	Male	Yes	7	Icelandic horse	G2	6	Yes
10	Male	Yes	19	Icelandic horse	G1	7	Yes
11	Male	Yes	19	Icelandic horse	G1	7	Yes
12	Male	Yes	6	Icelandic horse	G2	7	Yes
13	Male	Yes	12	Icelandic horse	G2	8	Yes
14	Male	Yes	10	Icelandic horse	G1	6	Yes
15	Male	Yes	6	Shetland pony	G2	6	No

Number, sex, age, breed, trial group (G1 and G2), assessment of body condition score (BCS), and the presence of cresty neck before diet supplementation of oligosaccharides or placebo in obese (BCS ≥ 5) horses are presented. Before entering the study, all horses were either diagnosed with Equine Metabolic Syndrome or had a phenotype to suspect it.

### 2.3 Study design

**Figure 1:**
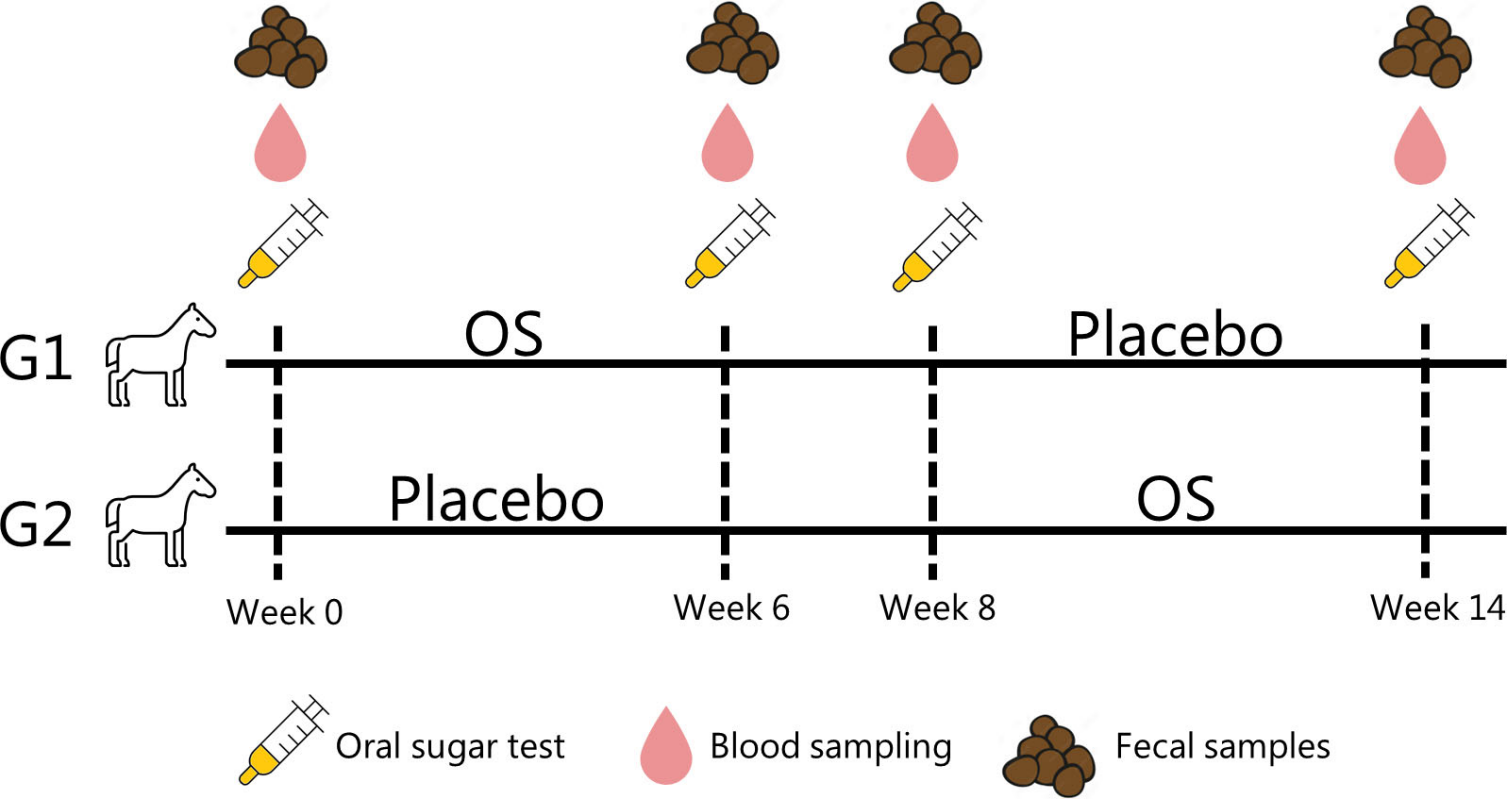
Cross-over trial with fifteen horses randomly assigned to two trial groups (G1 and G2). All horses had an oral sugar test (OST) performed at weeks 0, 6, 8, and 14. In the first trial period (P1), the horses were fed either an oligosaccharide feed supplementation (OS) or calcium carbonate (placebo). The period was followed by a two-week wash-out period. Subsequently, the horses were fed the other feed supplement in the second trial period (P2).

Horses were randomly grouped into two trial groups, G1 and G2, in a cross-over study. Eight horses were assigned to G1, and seven horses were assigned to G2 by a blinded extraction. Owners with more than one horse participating in the experiment had their horses equally divided into G1 and G2. All owners were blinded. Both groups followed the schedule in **Figure 1**. The study was conducted from September to January.

Following the cross-over design in **Figure 1**, the horses were either given Immulix® (Brogaarden ApS, Lynge, Denmark), a commercially available prebiotic feed supplement consisting of a proprietary blend of mannan-oligosaccharides and fructo-oligosaccharides (81.6 g/100 g of the supplement) referred to as OS (Lindenberg et al., 2021), or a placebo consisting of calcium carbonate. Owners fed horses the supplement once daily, either in a syringe mixed with water or as powder sprinkled over concentrate feed. With a cut-off at 250 kg body weight, the horses were either given 30 g (< 250 kg) or 50 g (> 250 kg) of the feed supplement. The placebo was given as 30 g regardless of weight.

Before and after each trial period (P1 and P2) an OST was performed. Before the OST, horses were housed overnight at the veterinary clinic and fasted for 10 to 12 hours. After fasting, horses were given 0.2 mL/kg body weight Dansukker glukosesirup (Nordic Sugar A/S, Copenhagen, Denmark) perorally with a syringe. Blood samples were collected intravenously from the jugular vein with vacutainers at 60 minutes for ponies or 75 minutes for horses after oral dosing (Smith et al., 2016; Frank and Walsh, 2017; Durham et al., 2019). The dosage of glucose provided 216 mg/kg body weight of saccharides.

### 2.4 Blood sample processing and analysis

Blood samples were collected into two 10 mL Lithium Heparin tubes from BD vacutainer® and one 10 mL serum Clot Activator Tube from BD Vacutainer® via jugular venipuncture. Immediately after blood collection, glucose was measured with a glucometer (FreeStyle Freedom Lite Blood Glucose Monitoring System, Abbott Diabetes Care Inc., Alameda, California, USA) with compatible blood glucose test strips (FreeStyle Lite Blood Glucose Test Strips, Abbott Diabetes Care Ltd., Oxon, United Kingdom) in whole blood collected from the side of the serum vacutainer. After blood collection, vacutainers were set to rest at ambient temperature for 30 minutes before centrifugation at 1,401 x g for 10 minutes with EICKEMEYER® Table Top Centrifuge. Afterward, all plasma and serum were harvested and kept at -20°C for one to three hours. Until analysis, endotoxin plasma samples were stored at -20°C, and the remaining samples were stored at -80°C. Insulin, SAA, and endotoxin concentrations in plasma and fecal diversity were measured in each sample from the OST for each animal.

### 2.5 ELISA

Plasma insulin was determined with a commercial equine-optimized ELISA (Mercodia Equine Insulin ELISA, Mercodia AB, Uppsala, Sweden) without further dilution of samples. PlasmaSAA was determined with a commercial equine-optimized ELISA (Horse Serum Amyloid A ELISA kit (SAA), Abclonal Inc., Woburn, Massachusetts, USA) without further dilution of samples. When measuring plasma SAA concentrations, 20 out of 60 samples were randomly selected and analyzed in duplicate (see **Appendix 4**). For plasma insulin concentrations, 24 out of 60 samples were randomly selected and analyzed in duplicate (see **Appendix 4**).

### 2.6 Endotoxin

Endotoxin concentrations were determined with a Pyrogene Recombinant Factor C Assay (PyroGene™ Recombinant Factor C Endotoxin Detection System, LONZA, Walkersville, Maryland, USA). Plasma was diluted with Limulus Amebocyte Lysate (LAL) in a 1:1000 dilution. Heating of diluted samples at 70°C for 10 minutes was added to the protocol before plating diluted samples. All endotoxin measurements were performed in duplicate.

### 2.7 Fecal sample processing

Fecal samples were collected into Biosphere® SafeSeal Tube 2.0 mL (Biosphere® SafeSeal Tube 2.0 mL, Sarstedt AG & Co. KG, Nümbrecht, Germany) before oral glucose dosing at every performed OST. The samples were immediately stored at -20°C for 1-3 hours. Subsequently, the samples were kept at -80°C until the time of analysis.

### 2.8 Sequencing of bacterial community in fecal samples

#### 2.8.1 DNA extraction

The genomic DNA was extracted using Bead-Beat Micro AX Gravity Kit (A&A Biotechnology, Gdynia, Poland) according to the manufacturer’s instruction. DNA concentration was determined by Qubit™ dsDNA High Sensitivity kot (MA, Unites States) and purity was measured using NanoDrop ND-1000 spectrophotometer (Saveen and Werner AB, Sweden).

#### 2.8.2 Library preparation and sequencing

A 16S rRNA gene amplicon library was constructed by amplifying the 16S rRNA gene with multiple forward and reverse primers targeting the 16S V1-V9 regions. PCR conditions for the amplification as follows: 95°C for 5 min, 2 cycles of 95°C for 20 s, 48°C for 30 s, 65°C for 10 s, 72°C for 45 s, and a final extension at 72°C for 4 min. A second PCR step was then performed to barcode PCR amplicons with the following conditions: 95°C for 2 min followed by 33 cycles of 95°C for 20 s, 55°C for 20 s, 72°C for 40 s, and a final extension at 72°C for 4 min. After each PCR reaction, PCR amplicons were cleaned up using SpeedBeads™ magnetic carboxylate (obtained from Sigma Aldrich). The size of barcoded PCR products (approximately 1500 bp) were checked by 1.5% agarose gel electrophoresis.

Sequencing libraries consisting of pooled barcoded PCR products from up to 196 samples were prepared by following the ligation sequencing protocol SQK-LSK110 (Oxford Nanopore Technologies, Oxford, UK) and loaded on R9.1.4 flow cell for 72 hours using GridIONX5 (Oxford Nanopore Technologies, Oxford, UK).

Nanopore sequencing software MinKNOW v. 2.0 (*GridION version 21.02.5)* was used for data collection (https://nanoporetech.com). Base calling and demultiplexing were performed by ONT’s Guppy v. 4.5.2 from Oxford Nanopore Technologies (https://nanoporetech.com).

For filtering and trimming of demultiplexed sequences Nanofilt v. 2.7.1. was used, with the filtering of data set to a minimum of 1000 and a maximum of 1600 reads, and a minimum average read quality score of 8 (see **Appendix 5**). Next, 15 nucleotides were trimmed from both ends of the reads, and the taxonomy was assigned by using the Parallel_assign_taxonomy_ucluster.py script of Quantitative Insigts into Microbial Ecology (Qiime) 1 v. 1.8.0. For reference database, Greengenes database v. 13.8 was used.

#### 2.8.3 Bioinformatic analysis

Initially, the dataset was purged for zOTU’s, which were detected in less than 5% of the samples, but the resulting dataset still maintained 98% of the total reads. Cumulative sum scaling (Paulson et al., 2013) was applied for the analysis of beta-diversity to counteract that a few zOTU’s represented a majority of count values, since cumulative sum scaling has been benchmarked with high accuracy for the applied metrics (Weiss et al., 2017). Cumulative sum scaling normalization was performed using the R software using the metagenomeSeq package (Paulson, 2014). To calculate the alpha diversity of the GM within horses in a group the weighted Shannon diversity index was used. To analyze the diversity between two groups, beta-diversity was used represented by Bray Curtis’s dissimilarity. Alpha-diversity analysis was based on raw read counts, rarefied to a median depth of 44574. R version 4.01 was used for subsequent analysis and presentation of data (R. C. Team, 2022). The main packages used were phyloseq (McMurdie and Holmes, 2013), vegan (Dixon, 2003), deseseq 2 (Love et al., 2014), ampvis2 (Andersen et al., 2018), ggpubr (Kassambara), and ggplot2 (Wickham, 2011). The data and code used are uploaded as **Supplementary Data**.

#### 2.8.4 Statistical analysis

All data were analyzed using GraphPad Prism version 9.0 for Mac (GraphPad Software Inc., San Diego, California, USA). Insulin, glucose, SAA, and endotoxin concentrations were compared using Δ-values (post/pre) of both feed supplements in a paired t-test. The bacteria with a relative abundance >1% were compared using Δ-abundances (post/pre) of both feed supplements in a Kruskal Wallis test divided by sex and feed supplement.

All parameters were analyzed and compared using the same procedure for sex (mare/gelding), prior laminitis diagnosis (previous laminitis/no laminitis), MH treatment status (treated/untreated), breed (warmblood/cold blood; Icelandic horses/remaining; Shetland ponies/remaining), and age (above mean age/under mean age). To compare two corresponding groups 2-way ANOVA was used. All data were considered significant if *p* ≤ 0.05.

## 3 Results

### 3.1 Reduced insulin concentrations in geldings

**Figure 2:**
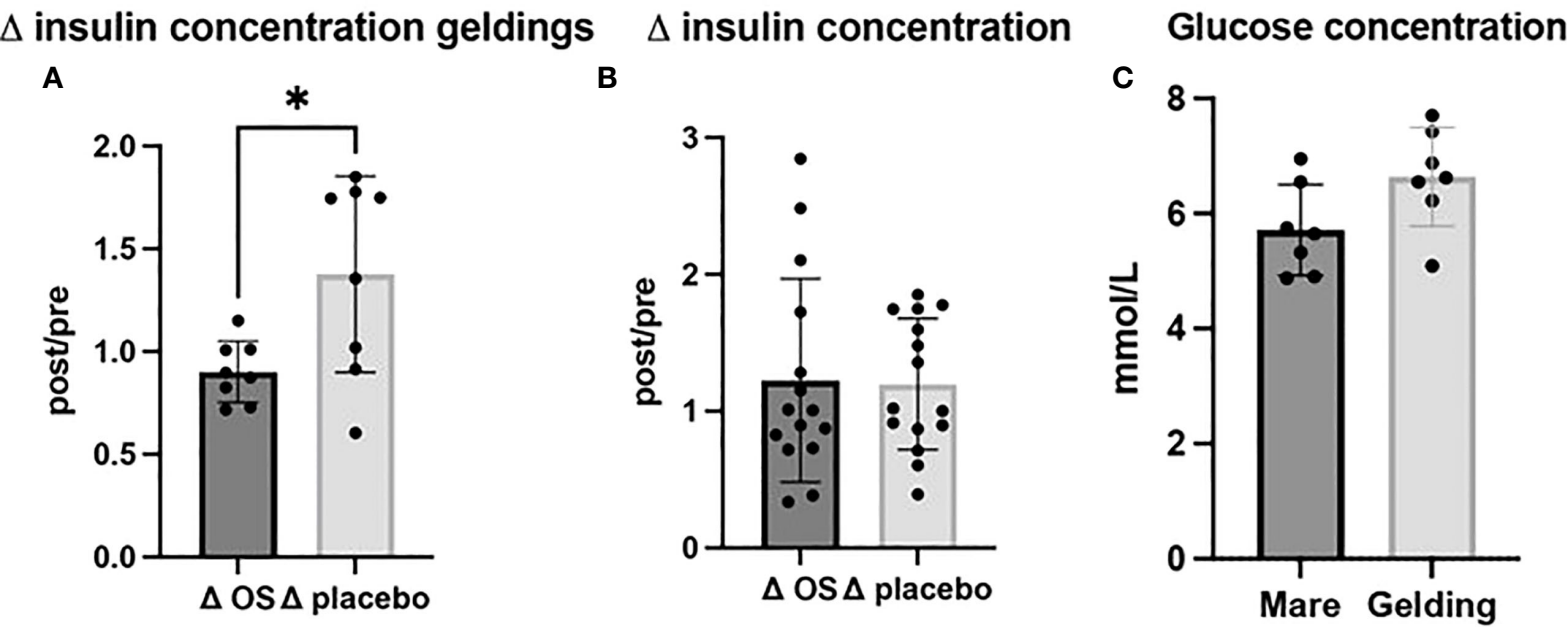
Cross-over study measuring plasma insulin and glucose concentrations in 15 horses, with a diagnosis of EMS or a veterinary history suspecting it, before (pre) and after (post) a six-week oligosaccharide feed supplement (OS) and placebo supplement during an oral sugar test (OST) using glucose at a dose of 0.2 mL/kg body weight. Data is presented as **(A)** *Δ* post-/pretreatment plasma insulin concentration in geldings, **(B)** *Δ* post-/pretreatment plasma insulin concentration of the overall study population, and **(C)** glucose concentrations of geldings and mares. * Significant p-value, *p* < 0.05.

Ten out of 15 horses had a plasma insulin concentration > 60 mIU/L at the beginning of the study, and thereby they exceeded the minimum concentration for diagnosing ID (Lindase et al., 2016). In geldings, ΔOS (post/pre) insulin concentrations were significantly reduced (*p* = 0.02) (n = 8) compared to Δplacebo (n = 8) (**Figure 2A**). On the contrary, glucose concentrations of geldings tended to be higher compared to mares (*p* = 0.059) (**Figure 2C**). In an overall evaluation including both sexes, there was no significant difference (*p* = 0.9) in plasma concentrations of insulin between treatment groups (**Table 2**; **Figure 2B**).

**Table 2:** Cross-over study values of whole blood (glucose) and plasma (insulin, SAA, and endotoxin concentrations) in 15 horses (geldings and mares), with a diagnosis of EMS or a veterinary history suspecting it, before (pre) and after (post) a six-week oligosaccharide feed supplement (OS) and placebo supplement during an oral sugar test (OST) using glucose at a dose of 0.2 mL/kg body weight.

Treatment	Pre	Post	Δ *p* value
Insulin (mIU/L)			
OS	96.2 ± 72.1	108.1 ± 91.0	0.93
Placebo	98.1 ± 86.0	104.4 ± 90.6	
Glucose (mmol/L)			
OS	5.8 ± 1.2	6.1 ± 1.1	0.059
Placebo	6.3 ± 1.1	6.3 ± 1.5	
SAA (ng/mL)			
OS	29.3 ± 21.4	25.0 ± 16.7	0.33
Placebo	35.4 ± 32.7	22.6 ± 13.1	
Endotoxin (EU/mL)			
OS	35.8 ± 35.2	29.9 ± 51.5	0.34
Placebo	54.5 ± 68.2	18.8 ± 31.4	

Data are presented as means ± standard deviation of insulin, glucose, SAA, and endotoxin concentrations. Adjusted sample p values of *Δ*post-/pretreatment p values are included. n = 15 for each treatment.

* Significant *p* value, *p* < 0.05; mIU/L, milli-international units per milliliter; EU/mL, Endotoxin Units per milliliter.

### 3.2 No significant effect on circulating secondary readouts

In the current study, circulating secondary readouts were measured to assess OS’s effects on metabolism and inflammation. **Table 3** depicts an overview of measured parameters. Data of mean values ± standard deviation of insulin, glucose, SAA, and endotoxin is presented. A tendency (*p* = 0.094) towards higher glucose concentrations was observed during placebo supplementation than during OS. Even so, no significant effect of OS was observed when comparing ΔOS and Δplacebo in all readouts.

**Table 3:** Cross-over study analyzing the relative abundance of the bacterias with a relative abundance > 1% in fecal samples of 15 horses, with a diagnosis of EMS or a veterinary history suspecting it, before (pre) and after (post) a six-week oligosaccharide feed supplement (OS) and placebo supplement.

Kingdom	Phylum	Class	Order	Family	Genus	Mares				Geldings			
						ΔOS	Δplacebo	*p* value	*q* value	ΔOS	Δplacebo	*p* value	*q* value
Bacteria	Actinobacteria	Coriobacteriia	Coriobacteriales	Coriobacteriaceae		1,45	2,63	0.9999	1.000	3,66	4,43	0.5781	0.8493
Bacteria	Firmicutes	Clostridia	Clostridiales			1,01	1,94	0.8438	0.9091	2,37	1,57	0.5781	0.8493
Bacteria	Firmicutes	Clostridia	Clostridiales	Clostridiaceae		0,27	1,25	0.6875	0.9091	0,26	0,80	0.1094	0.7997
Bacteria	Firmicutes	Clostridia	Clostridiales	Clostridiaceae	Clostridium	2,32	4,18	0.8438	0.9091	0,35	2,07	0.6875	0.8546
Bacteria	Firmicutes	Clostridia	Clostridiales	Lachnospiraceae		0,60	0,65	0.8438	0.9091	0,91	1,50	0.9375	0.9469
Bacteria	Firmicutes	Clostridia	Clostridiales	Ruminococcaceae		0,62	1,15	0.6875	0.9091	1,10	1,11	0.9375	0.9469
Bacteria	Firmicutes	Clostridia	Clostridiales	Ruminococcaceae	Ruminococcus	0,27	1,73	0.0938	0.9091	0,96	5,78	0.6875	0.8546
Bacteria	Firmicutes	Clostridia	Clostridiales	Veillonellaceae	Phascolarctobacterium	0,12	1,34	0.2188	0.9091	3,12	0,78	0.5781	0.8493
Bacteria	Firmicutes	Clostridia	Clostridiales	Mogibacteriaceae		0,88	2,30	0.5625	0.9091	4,06	0,97	0.2969	0.7997
Bacteria	Firmicutes	Erysipelotrichi	Erysipelotrichales	Erysipelotrichaceae	Unknown Erysipelotrichaceae	0,69	2,38	0.5625	0.9091	2,51	1,09	0.2969	0.7997
Bacteria	Firmicutes	Clostridia	Clostridiales	Clostridiaceae	Sarcina	0,70	0,65	0.3125	0.9091	0,11	6,14	0.1562	0.7997
Bacteria	Firmicutes	Clostridia	Clostridiales	Mogibacteriaceae	Mogibacterium	0,69	3,70	0.3125	0.9091	4,03	1,19	0.4688	0.8493
Bacteria	Firmicutes	Erysipelotrichi	Erysipelotrichales	Erysipelotrichaceae	Unknown Erysipelotrichaceae	0,69	2,38	0.5625	0.9091	2,51	1,09	0.2969	0.7997
Bacteria	Saccharibacteria	Unknown Saccharibacteria				0,69	2,49	0.5625	0.9091	3,32	1,69	0.2969	0.7997
Bacteria	Verrucomicrobia	Unknown Verrucomicrobia				1,02	1,73	0.4375	0.9091	1,80	1,22	0.5781	0.8493
Bacteria	Verrucomicrobia	Verrucomicrobiae	Verrucomicrobiales	Verrucomicrobiaceae	Akkermansia	7,05	10,73	0.8438	0.9091	0,87	3,84	0.8125	0.9379

Data is presented as Δ-abundance (post/pre) analyzed by a Kruskal Wallis test on OUTs from each sample. All presented differences are considered significant with a *q*-value < 0.05.

### 3.3 Tendency towards reduced Δ-endotoxin concentration in horses with previous laminitis

**Figure 3:**
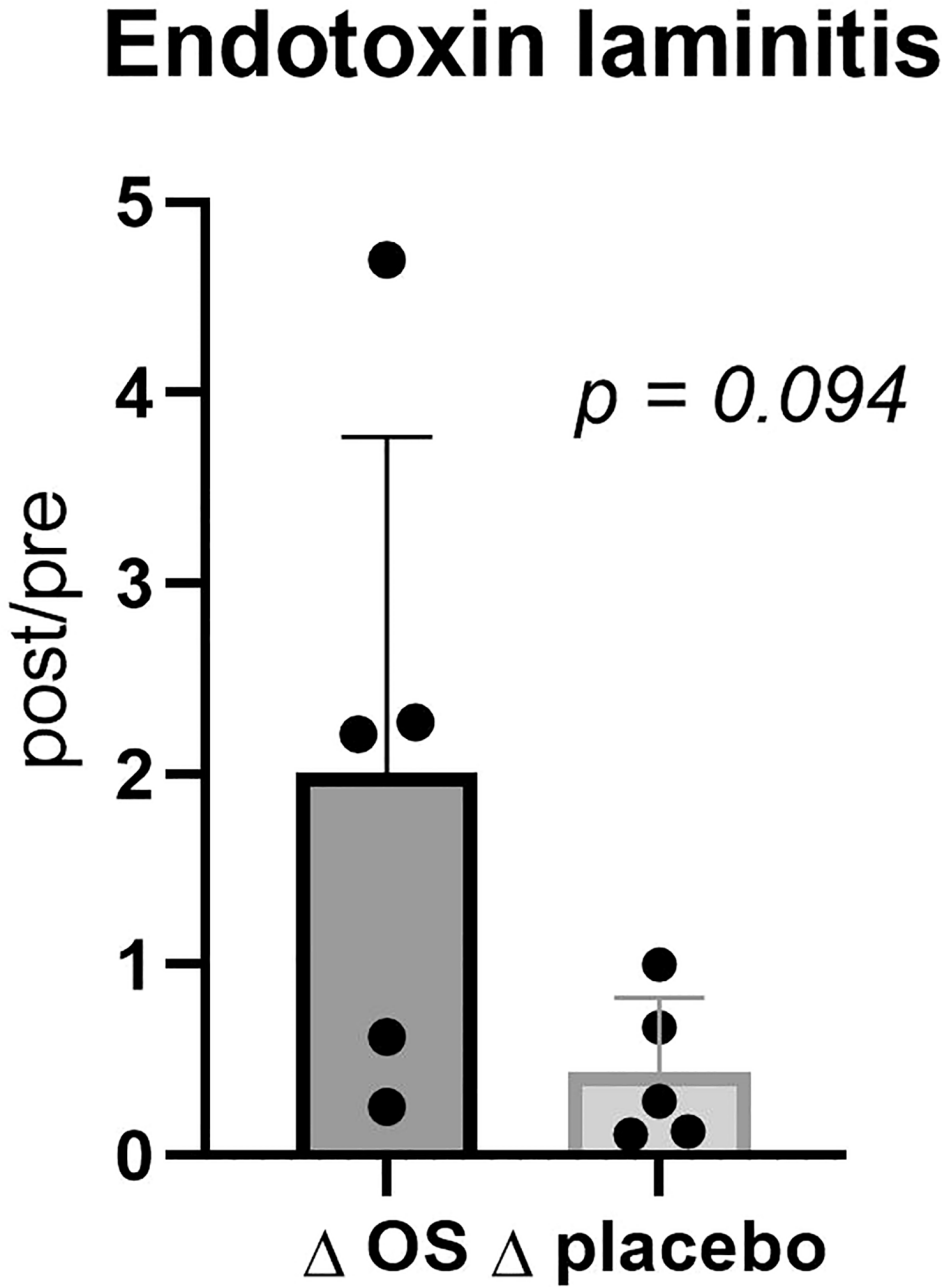
Cross-over study measuring endotoxin concentrations in five horses, with previous laminitis and a diagnosis of EMS or a veterinary history suspecting it, before (pre) and after (post) a six-week oligosaccharide feed supplement (OS) and placebo supplement during an oral sugar test (OST) using glucose at a dose of 0.2 mL/kg body weight. Data is presented as post-/pretreatment *Δ* of plasma endotoxin concentrations. Tending *p*-value, *p >*0.05 and <0.10.

A tendency towards a greater reduction in endotoxin concentration *Δ*placebo than *Δ*OS (post/pre) (*p* = 0.094) was discovered in horses with previous laminitis (**Figure 3**). However, no significant difference was found in endotoxin concentration in the overall study population (*p* = 0.3).

### 3.4 No influence of breed or age on the effect of oligosaccharide supplementation

Increased susceptibility to ID with increased age (Nielsen et al., 2010; Morgan et al., 2014; Hart et al., 2016; Rapson et al., 2018) and in specific breeds (Pleasant et al., 2013; Suagee et al., 2013) has been suggested. Therefore, we investigated if these factors would influence the effect of OS. However, there was no significant influence of breed or age on the effect of OS in any circulating readouts.

### 3.5 Bacterial abundances affected by diet, insulin concentration, and laminitis status

In the overall study population, none of the bacterias changed significantly in relative abundance through ΔOS or Δplacebo supplementation (see **Table 3**).

**Figure 4:**
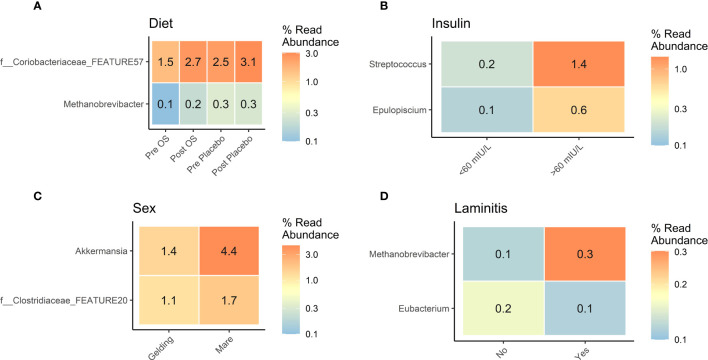
Cross-over study analyzing the differential abundance of gut microbiota in fecal samples of 15 horses, with a diagnosis of EMS or a veterinary history suspecting it, before (pre) and after (post) a six-week oligosaccharide feed supplement (OS) and placebo supplement. Data is presented as differential abundance grouped by **(A)** diet (pre OS, post OS, pre placebo, and post placebo), **(B)** insulin concentration < 60 mIU/L/> 60 mIU/L), **(C)** sex (gelding/mare), and **(D)** laminitis status (“no” no laminitis/”yes” previous laminitis). The differential abundance is presented as percent read abundance. Comparisons were performed by group, and for diet the comparison was performed between the Pre OS/placebo and Post OS/placebo groups. All displayed taxa were differentially abundant p-value < 0.05 and log2 fold change >2.

The influence of sex on insulin concentrations inspired to analyze the GM considering diet, insulin concentrations, sex, and laminitis status. At post OS and post placebo, the *Coriobacteriaceae* family was significantly more abundant than in pre OS and pre placebo. Furthermore, the *Methanobrevibacter* genus was significantly more abundant at pre placebo and post placebo than in pre OS and post OS (**Figure 4A**).

In horses with insulin concentrations > 60 mIU/L, the differential abundance of *Streptococcus* genus was sevenfold greater and *Epuloscium* genus was sixfold greater than in horses with insulin concentrations < 60 mIU/L (**Figure 4B**).

Mares had a significantly greater differential abundance of the *Akkermansia* genus and Clostridiaceae family than geldings (**Figure 4C**).

The five horses with a previous diagnosis of laminitis had a greater abundance of *Methanobrevibacter* genus, whereas the *Eubacterium* genus was significantly more abundant in horses without previous laminitis (**Figure 4D**).

### 3.6 Significantly greater Shannon diversity in horses with high insulin concentrations

**Figure 5:**
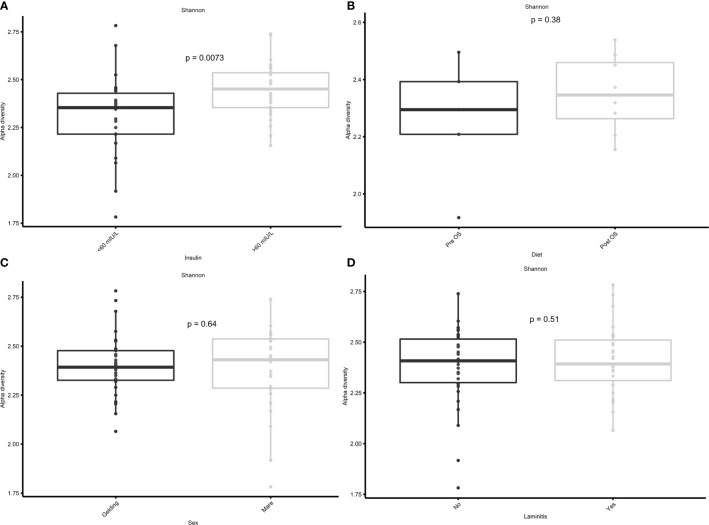
Cross-over study analyzing the Shannon diversity in fecal samples of 15 horses, with a diagnosis of EMS or a veterinary history suspecting it, before (pre) and after (post) a six-week oligosaccharide feed supplement (OS) and placebo supplement. Data is grouped by **(A)** before and after oligosaccharide feed supplementation, **(B)** insulin concentrations > 60 mIU/L/< 60 mIU/L, **(C)** sex (gelding/mare), and **(D)** previous diagnosis of laminitis (“yes”/”no”). Data are presented as a minimum, lower quartile, median, upper quartile, and maximum. Significant p-value, *p* < 0.05.

In the current study, the Shannon diversity of the GM in horses with insulin concentrations > 60 mIU/L was significantly greater (*p* = 0.0073) than in horses with insulin concentrations < 60 mIU/L (**Figure 5A**). However, no significant difference was found in Shannon diversity between pre/post OS (*p* = 0.38), sex (*p* = 0.64), and laminitis status (*p* = 0.51) (**Figures 5B–D**).

### 3.7 No significant change in Shannon diversity after oligosaccharide diet supplementation

**Figure 6:**
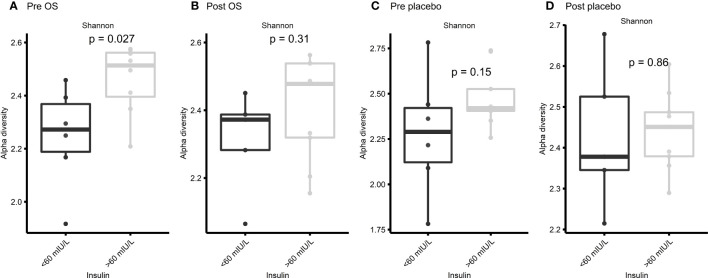
Cross-over study analyzing the Shannon diversity in fecal samples of 15 horses, with a diagnosis of EMS or a veterinary history suspecting it, before (pre) and after (post) a six-week oligosaccharide feed supplement (OS) and placebo supplement. The figures show insulin concentrations > 60 mIU/L/< 60 mIU/L at **(A)** pre OS, **(B)** post OS, **(C)** pre placebo and **(D)** post placebo. Data are presented as a minimum, lower quartile, median, upper quartile, and maximum. Significant p-value, *p* < 0.05.

The significant difference in Shannon diversity between horses with insulin concentration > 60 mIU/L and < 60 mIU/L inspired to specify the Shannon diversity analysis into pre OS, post OS, pre placebo, and post placebo regarding insulin concentration. No significant difference was observed between the two groups at post OS (*p* = 0.31), pre placebo (*p* = 0.15), and post placebo (*p* = 0.86) (**Figures 6B–D**). However, a significant difference (*p* = 0.027) was observed at pre OS (**Figure 6A**).

### 3.8 Beta diversity dependent on insulin concentration and laminitis status

**Figure 7:**
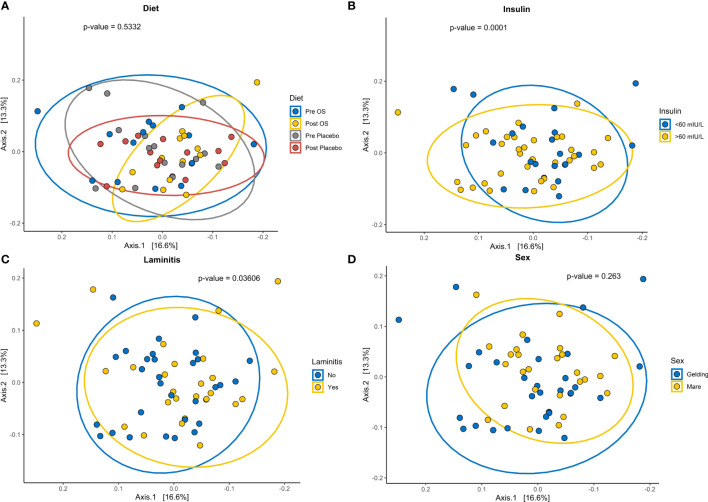
Cross-over study analyzing the beta diversity in fecal samples of 15 horses, with a diagnosis of EMS or a veterinary history suspecting it, before (pre) and after (post) a six-week oligosaccharide feed supplement (OS) and placebo supplement. The figures show beta diversity of **(A)** before (pre OS) and after (post OS) a six-week oligosaccharide feed supplementation, and before and after placebo (pre and post placebo), **(B)** insulin concentration > 60 mIU/L and < 60 mIU/L “no”, **(C)** previously diagnosed with laminitis “yes” or not previously diagnosed with laminitis “no”, and **(D)** sex (geldings and mares). Significant p-value, p < 0.05. Permutation Based Analysis of Variance (PERMANOVA) was used for calculation differences between group Bray-Curtis dissimilarities.

The GM beta diversity analysis of the 15 horses grouped into insulin concentrations > 60 mIU/L and insulin concentrations < 60 mIU/L showed a significantly difference in community composition of horses with insulin concentrations > 60 mIU/L (*p* = 0.0002) (**Figure 7B**). Furthermore, a comparison of horses with previous laminitis “yes” to horses without previous laminitis “no” showed a significant difference in community composition (**Figure 7C**). Even so, no significant difference in bacterial composition was observed before and after oligosaccharide feed supplementation and placebo supplementation and sex (**Figures 7A, D**).

## 4 Discussion

In this population of horses previously diagnosed with or matching the phenotype for EMS, we observed a significant reduction in *Δ*-insulin concentrations in geldings fed OS, while no significance was found in mares. Additionally, no significant effect was observed in SAA and endotoxin concentrations. Respondek et al. (2011) found that sc-FOS increased SI in eight Arabian geldings with insulin resistance (Respondek et al., 2011). However, McGowan et al. (2013) investigated the effect of dietary restriction in combination with sc-FOS on SI in 10 mares and two geldings diagnosed with EMS of different breeds and discovered no significant effect of sc-FOS (Respondek et al., 2011; McGowan et al., 2013). This could indicate that the effect of sc-FOS on ID is influenced by sex. Nevertheless, Pleasant et al. (2013) investigated the correlation between insulin, glucose, and sex and found no correlation in healthy horses, while still finding a significant decrease in SI with increasing age in mares but not geldings (Pleasant et al., 2013). Additionally, significantly higher insulin concentrations have previously been observed in mares (Valenzuela et al., 2017), which contrasts with the current study in which glucose concentrations tended to be higher in geldings compared to mares. The higher glucose concentrations in geldings were not accompanied by higher insulin concentrations when compared to mares. This might be explained by a lower relative insulin response to glucose in geldings (Wilcox, 2005). Sex’s influence on the effect of OS is supported by the literature. In both humans and horses, sex has been shown to influence GM composition, disease susceptibility, and the presence of sex hormones, such as estrogen (Whitacre, 2001; Fish, 2008; Haro et al., 2016; vom Steeg and Klein, 2016; Hu et al., 2020). The latter has been shown to induce increased cytokine production of interleukin 1, interleukin 10, and interferon-γ by immune cells (vom Steeg and Klein, 2016). In the analysis of the development of bacterial abundances in mares and geldings, no significant changes were found. This leads to the question if the impact of GM is caused by several minor changes in microbial abundances and not a few significant changes.

In mice it has been shown that the same mice line from two different breeders and, thereby with essential differences in GM, respond very differently to anti-diabetic treatment (Bondarenko et al., 2022). No significant influence of sex was discovered in the weighted bacterial richness of the GM, Shannon diversity or the overall community composition. Also, the influence of the hormonal cycle in mares might have affected the results. Even so, since the higher glucose concentrations in geldings were not significant, more studies are needed to conclude on a difference in insulin response between sexes. In the current study, the diets of the horses, training intensity and pastor time were kept the same throughout the experiment (see **Appendix 2**). Nevertheless, the horses were fed, housed, and exercised very differently, which might have diminished the actual effect of OS.

In the present study the horses were fed either 40.8 g OS/daily or 24.48 g OS/daily. When comparing that to the daily FOS dosage of McGowan et al. (2013) 10 g FOS/100 kg/daily and Respondek et al. (2011) (45 g FOS/daily) it is comparable to the latter. As the period with OS supplementation is the same, the different results in McGowan et al. (2013) indicate that low doses of OS could explain the nonsignificant results (Respondek et al., 2011; McGowan et al., 2013). Further, crossover study design in Respondek et al. (2011) varies from the present, as it contains 21 days of wash out period. However, as similar studies have implemented wash out periods between 7 and 28 days, a wash-out period of 14 days was chosen (Respondek et al., 2011; Costa et al., 2015; Fernandes et al., 2021; Arias-Esquivel et al., 2023).

The observed differences might not be related to the composition of the GM and conflicting results of OS supplementation might be a consequence of different breeds and ages of horses in the respective study populations (Treiber et al., 2006; Pleasant et al., 2013; Bamford et al., 2016). However, we were unable to confirm previous studies revealing age and breed-related susceptibilities. Except for pre-OS, no significant differences in Shannon diversity regarding insulin concentrations were discovered. A subconsciously altered focus on the diet by the owners of the horses after study initiation might have caused this difference.

Metformin hydrochloride is used in horses with diagnosed EMS and/or in horses suffering from recurrent laminitis. In the current study, five out of 15 horses in the trial were treated orally with this antidiabetic drug and four of these horses were previously diagnosed with laminitis. In humans, MH acts systemically by inhibiting gluconeogenesis, decreasing hepatic glucose production, and increasing glucose transporter type 4 transporters on the cell membranes of skeletal muscle cells (Frank et al., 2010). However, the oral bioavailability of MH in horses is poor (3.9-7.1%) (Frank et al., 2010). In horses, MH’s effects on the insulin response are indirect and caused by decreased enteric glucose absorption (Rendle et al., 2013). It has been shown in mice that MH treatment can change the microbial composition of the gut with a significantly increased level of *Akkermansia* spp. in treated mice. Increasing the abundance of *Akkermansia* spp. significantly improved glucose tolerance and decreased adipose tissue (Shin et al., 2014). Hence, an altered GM might explain the decreased enteric glucose absorption in horses. An increase in *Akkermansia muciniphila* and other beneficial bacteria of the gut increases the production of short-chain fatty acids (Lukovac et al., 2014; Dao et al., 2016), which were positively correlated to the anti-inflammatory interleukin-10 and decreased systemic inflammation in horses (Lindenberg F. et al., 2019; Lindenberg et al., 2021). Thus, MH treatment might have affected the current results, with an increased anti-inflammatory response reducing the pro-inflammatory cytokine secretion by the adipose tissue in obese horses (Burns et al., 2010). Nevertheless, studies solely focusing on the inflammatory response and alterations of GM during MH treatment are needed to confirm these assumptions.

The suggested alteration of GM composition potentially has been influenced by laminitis status. In the current study, the bacterial community composition in horses with previous laminitis was significantly different from horses without laminitis. Steelman et al. (2012) discovered a change in the GM composition of horses with chronic laminitis when compared to healthy horses (Steelman et al., 2012).

The owners of previous laminitic horses were feeding high quality and low sugar feed and roughage to avoid hyperinsulinemia, which is the main reason for laminitis (Argo et al., 2015). Neves et al. (2020) discovered that a chronic high-sugar intake altered the GM in rats (de Oliveira Neves et al., 2020). A low sugar diet of high quality might have resulted in a more diverse GM when compared to horses who might have been fed a low-quality standard feed with higher sugar concentrations. With the connection between high insulin concentrations and the occurrence of laminitis in mind, the significantly greater GM Shannon diversity and community composition between horses with insulin concentrations > 60 mIU/L and < 60 mIU/L might have been caused by a difference in diet quality or MH treatment as already stated. Thus, studies feeding the same diet to all groups are needed to evaluate the effect of OS combined with MH and laminitis on GM. The differential abundance of the GM in horses with and without laminitis showed a significantly greater abundance of the *Eubacterium* genus in horses without laminitis. In humans, the *Eubacterium* genus is more abundant in women with metabolic diseases (Chan, 2013), supporting the theory of an altered GM in horses with laminitis and hyperinsulinemia. Also, a significantly increased abundance of *Akkermansia* genus and Clostridiaceae order was found in mares when compared to geldings. Both bacterial groups are positively correlated with an anti-inflammatory cytokine response (Lindenberg F. et al., 2019) and *Akkermansia* genus is negatively correlated with plasma glucose and positively correlated with SI in humans (Dao et al., 2016). This is in line with our findings that the plasma glucose concentrations of mares tended to be lower than in geldings. Even so, no significant difference in the differential abundance of *Akkermansia* genus was found in horses with < 60 mIU/L insulin concentration and the Verrucomicrobia phyla have been positively associated with obesity in horses diagnosed with EMS (Biddle et al., 2018). The *Streptococcus* and *Epuloscium* genera were significantly more abundant in horses with >60 mIU insulin concentration. However, to the author’s knowledge, neither of the genera is confounding with equine immunity, obesity, or ID. In humans, it is well known that oligosaccharides target *Bifidobacterium* spp (Mills et al., 2023), and this is also the case in mouse studies in case the mice harbor *Bifidobacterium* spp (Hansen et al., 2013). However, in horses, *Bifidobacterium* spp. are less common as gut inhabitants, and, therefore, these oligosaccharides target other species (Lindenberg et al., 2021); a phenomenon also observed in mice not harboring *Bifidobacterium* spp (Laigaard et al., 2020). tendencyHorses with previous laminitis tended to have a lower Δ-endotoxin concentration after placebo. Calcium is predominantly absorbed in the small intestines in horses (Sprekeler et al., 2011). However, in obese mice, dietary calcium was shown to affect the GM composition [115], resulting in lower plasma endotoxin levels (Chaplin et al., 2016). It is suggested that changes in the GM with a decrease of, e.g., Escherichia coli or improvement of mucosal barrier function might decrease the absorption of lipopolysaccharides and reduce inflammation and insulin resistance associated with low-grade endotoxemia (Berg et al., 2005; Cani et al., 2007; Respondek et al., 2007; Respondek et al., 2008). To the authors’ knowledge, this has not been shown in horses. Previous studies have investigated the impact of calcium carbonate on body weight and glucose tolerance. Nevertheless, none of the studies found any effect of calcium supplementation on body weight in equine clinical studies (Reid et al., 2005; Yanovski et al., 2009).

The GM composition and imbalance have been associated with diseases in both humans and horses. This study demonstrated an improvement from OS on insulin concentrations in geldings and alterations of the GM in specific groups of horses. Nevertheless, more extensive studies are needed to establish why some horses benefitted from OS while others did not. This might lead us closer to unraveling the connection between the GM, immune response, and insulin metabolism in EMS. The results of the present study are promising and suggest that OS could be a non-invasive way of improving health in horses.

## Data availability statement

The generated datasets for this study can be found in the Open Science Framework, https://osf.io/fqu9s/?view_only=5499d9d6ae694e3490115f72fd41ce15.

## Ethics statement

The animal study was reviewed and approved by Animal Ethics Institutional Board, Department of Veterinary and Animal Sciences, Faculty of Health and Medical Sciences, University of Copenhagen. Written informed consent was obtained from the owners for the participation of their animals in this study.

## Author contributions

AH and FL generated the idea. AM, SY, FL, and AH planned the experiments. AM and SY did all experimental procedures under the guidance of AH, FL, and NL. AM wrote the first draft of the manuscript, which hereafter was accepted by all authors. All authors contributed to the article and approved the submitted version.
