# Supplementary material for: Oligosaccharide feed supplementation reduces plasma insulin in geldings with Equine Metabolic Syndrome
Source: Front Microbiomes. 2023 Aug 2;2:1194705. doi: 10.3389/frmbi.2023.1194705 (PMC12993584; doi:10.3389/frmbi.2023.1194705)
Supplement: Supplementary file 1 [file DataSheet_1.zip › Appendix 1.PDF]

Appendix 1: Animal housing

Table 1: Fifteen horses included in a cross-over study at week 0. Horse number, sex, age, breed, trial group (G1 and G2), assessment of body condition score (BCS), and the presence of cresty neck before diet supplementation of oligosaccharides or placebo in obese (BCS $\geq$ 5) horses are presented. Before entering the study, all horses were either diagnosed with Equine Metabolic Syndrome or had a phenotype to suspect it.

Number	Sex	Gelding	Age (years)	Breed	Group	BCS (1-9)	Cresty neck (yes/no)
1	Female	-	12	Fjord horse	G2	7	Yes
2	Female	-	7	Icelandic horse	G1	6	Yes
3	Female	-	9	Shetland pony	G1	6	Yes
4	Female	-	3	Frederiksborg horse	G1	8	Yes
5	Female	-	5	Frederiksborg horse	G2	8	Yes
6	Female	-	16	Shetland pony	G2	7	Yes
7	Female	-	18	Shetland pony	G1	6	Yes
8	Male	Yes	14	Cross-breed	G1	7	Yes
9	Male	Yes	7	Icelandic horse	G2	6	Yes
10	Male	Yes	19	Icelandic horse	G1	7	Yes
11	Male	Yes	19	Icelandic horse	G1	7	Yes
12	Male	Yes	6	Icelandic horse	G2	7	Yes
13	Male	Yes	12	Icelandic horse	G2	8	Yes
14	Male	Yes	10	Icelandic horse	G1	6	Yes
15	Male	Yes	6	Shetland pony	G2	6	No

Horse number 1

Horse number 1 is stabled outdoor on a field during daytime and indoor in a box during nighttime. Prior to the study, it was diagnosed with EMS and is treated with MH. It is fed with low sugar concentrates three times a day, 4-5 kg hay, and additionally straw daily.

Horse number 2:

Horse number 2 is stabled outdoor in loose housing. Prior to the study, it has been diagnosed with EMS and is treated with MH. It is fed hay and vitamins daily.

Horse number 3:

Horse number 3 is stabled outdoor in a loose housing on a soil paddock except for three hours a day, where it is kept on a grass field with a mouth basket on. It is fed with a small dose of low sugar concentrates and in addition roughage.

Horse number 4:

Horse number 4 is stabled outdoor on grass fields during daytime and indoor in a box during nighttime. It is fed with concentrates and lucerne and in addition 3 kg hay during nighttime, and 1 kg hay and 2 kg straw during daytime.

Horse number 5:

Horse number 5 is stabled outdoor on grass fields during daytime and indoor in a box during nighttime. It is fed with concentrates and lucerne and in addition 3 kg hay during nighttime, and 1 kg hay and 2 kg straw during daytime.

Horse number 6:

Horse number 6 is stabled outside in loose housing on a grass paddock. It is fed with low-sugar concentrates and hay daily.

Horse number 7:

Horse number 6 is stabled outside in loose housing on a grass paddock. It is fed with low-sugar concentrates and hay daily.

Horse number 8:

Horse number 8 is stabled outside on a soil paddock during daytime and inside in a box during nighttime. Prior to the study, it has been diagnosed with EMS and is treated with MH. It is fed with low sugar concentrates and 16 kg roughage daily.

Horse number 9:

Horse number 9 is stabled outdoor in loose housing on a grass paddock. Prior to the study, it was diagnosed with EMS and is treated with MH. It is fed concentrates and roughage daily.

Horse number 10:

Horse number 10 is stabled outdoor in loose housing on a grass paddock. It is fed concentrates and roughage daily.

Horse number 11:

Horse number 11 is stabled outdoor on a paddock during daytime and indoor in a box during nighttime. It is fed concentrates and low-sugar hay daily.

Horse number 12:

Horse number 12 is stabled outside in loose housing. The paddock is primarily a soil paddock but there is a small amount of grass available. Prior to the study, it has been diagnosed with EMS. It is fed 6-7 kg soaked hay and vitamins daily.

Horse number 13:

Horse number 13 is stabled outside on a soil paddock. It suffers from a hard cresty neck whenever fed with grass. It is fed concentrates and hay daily.

Horse number 14:

Horse number 14 is stabled outside in loose housing. The paddock is primarily a soil paddock but there is a small amount of grass available. It is fed 6-7 kg soaked hay and vitamins daily.

Horse number 15:

Horse number 15 is stabled outdoor in loose housing on a soil paddock except for three hours a day, where it is kept on a grass field with a mouth basket on. Prior to the study, it was diagnosed with Pituitary Pars Intermedia Dysfunction, Equine Metabolic Disease, and laminitis. Therefore, it is

treated with metformin hydrochloride. It is fed with a small dose of low sugar concentrates and in addition roughage.
