# Supplementary material for: Oligosaccharide feed supplementation reduces plasma insulin in geldings with Equine Metabolic Syndrome
Source: Front Microbiomes. 2023 Aug 2;2:1194705. doi: 10.3389/frmbi.2023.1194705 (PMC12993584; doi:10.3389/frmbi.2023.1194705)
Supplement: Supplementary file 1 [file DataSheet_1.zip › Appendix 2.PDF]

Appendix 2: Body weight

	Weight (kg)			
Horse number	Pre OS	Post OS	Pre placebo	Post placebo
1	465	471	483	485
2	300	291	291	281
3	178	170	170	175
4	640	655	654	652
5	548	560	560	544
6	414	413	411	418
6	214	212	218	216
7	224	213	211	210
8	628	629	630	637
9	407	410	406	405
10	412	405	407	403
11	396	391	394	392
13	347	344	353	342
14	384	377	373	364
15	138	133	138	130
