# Supplementary material for: Oligosaccharide feed supplementation reduces plasma insulin in geldings with Equine Metabolic Syndrome
Source: Front Microbiomes. 2023 Aug 2;2:1194705. doi: 10.3389/frmbi.2023.1194705 (PMC12993584; doi:10.3389/frmbi.2023.1194705)
Supplement: Supplementary file 1 [file DataSheet_1.zip › Appendix 3.PDF]

Appendix 3

Plate overview - insulin:

	1	2	3	4	5	6	7	8	9	10	11	12
A	C0	C0	H1 W0	H8 W0	H10 W0	H12 W0	H13 W0	H3 W0	H5 W0	H1 P4	H12 P1	H3 W6
B	C1	C1	H1 W6	H8 W6	H10 W6	H12 W6	H13 W6	H3 W6	H5 W6	H2 P3	H12 P2	H3 W8
C	C2	C2	H1 W8	H8 W8	H10 W8	H12 W8	H13 W8	H3 W8	H5 W8	H8 P1	H14 P2	H4 W6
D	C3	C3	H1 W14	H8 W14	H10 W14	H12 W14	H13 W14	H3 W14	H5 W14	H8 P4	H13 P2	H4 W14
E	C4	C4	H2 W0	H9 W0	H11 W0	H14 W0	H15 W0	H4 W0	H6 W0	H10 P2	H15 P1	H5 W6
F	C5	C5	H2 W6	H9 W6	H11 W6	H14 W6	H15 W6	H4 W6	H6 W6	H10 P4	H15 P2	H6 W8
G	H7 W0	H7 W8	H2 W8	H9 W8	H11 W8	H14 W8	H15 W8	H4 W8	H6 W8	H11 P3	H15 W14	H6 W14
H	H7 W6	H7 W14	H2 W14	H9 W14	H11 W14	H14 W14	H15 W14	H4 W14	H6 W14	H11 P4	H3 W0	H7 W8

Plate overview - SAA:

	1	2	3	4	5	6	7	8	9	10	11	12
A	Std 500 pg	Std 500 pg	H1 W0	H8 W0	H10 W0	H12 W0	H13 W0	H3 W0	H5 W0	H7 W0	H10 W8	H13 W14
B	Std 250 pg	Std 250 pg	H1 W6	H8 W6	H10 W6	H12 W6	H13 W6	H3 W6	H5 W6	H7 W6	H10 W14	H15 W0
C	Std 125 pg	Std 125 pg	H1 W8	H8 W8	H10 W8	H12 W8	H13 W8	H3 W8	H5 W8	H7 W8	H11 W6	H15 W8
D	Std 62,5 p	Std 62,5 p	H1 W14	H8 W14	H10 W14	H12 W14	H13 W14	H3 W14	H5 W14	H7 W14	H11 W8	H3 W0
E	Std 31,2 p	Std 31,2 p	H2 W0	H9 W0	H11 W0	H14 W0	H15 W0	H4 W0	H6 W0	H2 W0	H11 W14	H3 W8
F	Std 15,6 p	Std 15,6 p	H2 W6	H9 W6	H11 W6	H14 W6	H15 W6	H4 W6	H6 W6	H2 W8	H12 W6	H4 W6
G	Std 7,8 pg	Std 7,8 pg	H2 W8	H9 W8	H11 W8	H14 W8	H15 W8	H4 W8	H6 W8	H9 W8	H14 W0	H4 W14
H	Std 0 pg/n	Std 0 pg/n	H2 W14	H9 W14	H11 W14	H14 W14	H15 W14	H4 W14	H6 W14	H10 W6	H13 W0	H5 W6

H = Horse
W = Week
Duplicates
Standardcurve

H = Horse
W = Week
Duplicates
Standardcurve
