# Supplementary material for: Oligosaccharide feed supplementation reduces plasma insulin in geldings with Equine Metabolic Syndrome
Source: Front Microbiomes. 2023 Aug 2;2:1194705. doi: 10.3389/frmbi.2023.1194705 (PMC12993584; doi:10.3389/frmbi.2023.1194705)
Supplement: Supplementary file 1 [file DataSheet_1.zip › Appendix 5.PDF]

Appendix 5: Clinical evaluation at week 0

Horse 1:

Body condition score 7/9.

No digital pulse. No heat in hooves.

Horse 2:

Body condition score 6/9.

No digital pulse. No heat in hooves.

Horse 3:

Body condition score 6/9.

No digital pulse. Slightly warm hooves. Increased abdominal fat.

Horse 4:

Body condition score 8/9.

No digital pulse. No heat in hooves. Cyathostomin parasites found in feces and treatment is initiated.

Horse 5:

Body condition score 8/9.

No digital pulse. No heat in hooves.

Horse 6:

Body condition score 7/9.

No digital pulse. No heat in hooves.

Horse 7:

Body condition score 6/9.

No digital pulse. No heat in hooves.

Horse 8:

Body condition score 7/9.

No digital pulse. No heat in hooves.

Horse 9:

Body condition score 6/9.

No digital pulse. No heat in hooves.

Horse 10:

Body condition score 7/9.

No digital pulse. No heat in hooves.

Horse 11:

Body condition score 7/9.

No digital pulse. No heat in hooves. Decreased muscle mass and increased abdominal fat.

Horse 12:

Body condition score 7/9.

No digital pulse. No heat in hooves.

Horse 13:

Body condition score 7-8/9.

No digital pulse. No heat in hooves.

Horse 14:

Body condition score 6/9.

No digital pulse. No heat in hooves.

Horse 15:

Body condition score 6/9.

Little or no digital pulse. Slightly warm hooves. Decreased muscle mass and increased abdominal fat.
