# Supplementary figures and images for: Oligosaccharide feed supplementation reduces plasma insulin in geldings with Equine Metabolic Syndrome
Source: Front Microbiomes. 2023 Aug 2;2:1194705. doi: 10.3389/frmbi.2023.1194705 (PMC12993584; doi:10.3389/frmbi.2023.1194705)

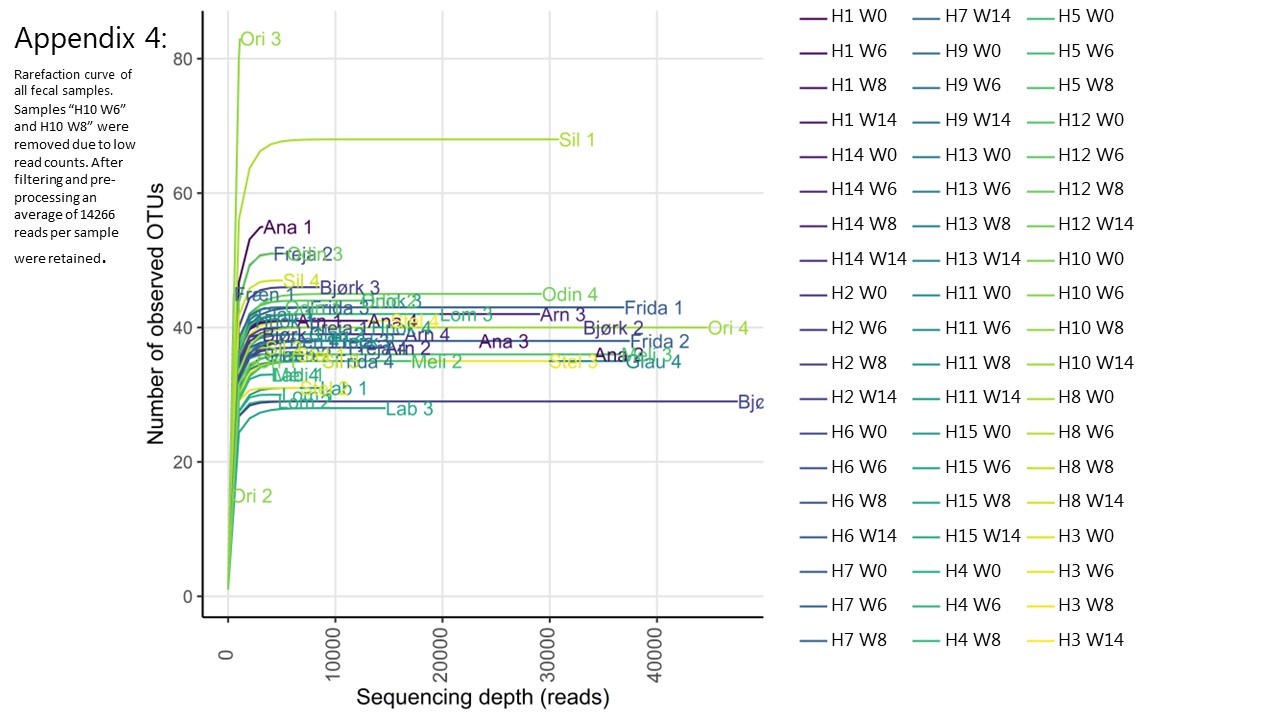

Supplement: Supplementary file 1 [file DataSheet_1.zip › Appendix 4.JPEG]
